# DRGAN: Dense Residual Generative Adversarial Network for Image Enhancement in an Underwater Autonomous Driving Device

**DOI:** 10.3390/s23198297

**Published:** 2023-10-07

**Authors:** Jin Qian, Hui Li, Bin Zhang, Sen Lin, Xiaoshuang Xing

**Affiliations:** 1 College of Information Engineering, Taizhou University, Taizhou 225300, China; huili_tzxy@tzu.edu.cn (H.L.); zhangbin@tzu.edu.cn (B.Z.); 2 School of Automation and Electrical Engineering, Shenyang Ligong University, Shenyang 110159, China; lin_sen6@126.com; 3 School of Computer Science and Engineering, Changshu Institute of Technology, Changshu 215506, China; xing@cslg.edu.cn

**Keywords:** deep learning, underwater autonomous driving device, underwater image enhancement, generative adversarial network

## Abstract

Underwater autonomous driving devices, such as autonomous underwater vehicles (AUVs), rely on visual sensors, but visual images tend to produce color aberrations and a high turbidity due to the scattering and absorption of underwater light. To address these issues, we propose the Dense Residual Generative Adversarial Network (DRGAN) for underwater image enhancement. Firstly, we adopt a multi-scale feature extraction module to obtain a range of information and increase the receptive field. Secondly, a dense residual block is proposed, to realize the interaction of image features and ensure stable connections in the feature information. Multiple dense residual modules are connected from beginning to end to form a cyclic dense residual network, producing a clear image. Finally, the stability of the network is improved via adjustment to the training with multiple loss functions. Experiments were conducted using the RUIE and Underwater ImageNet datasets. The experimental results show that our proposed DRGAN can remove high turbidity from underwater images and achieve color equalization better than other methods.

## 1. Introduction

With developments in science and technology, Internet of Things (IoT) technology has been introduced into advanced underwater vision tasks, such as autonomous underwater driving, ocean scene analysis, and fisheries. Underwater data, such as the marine environment, marine density, and seafloor pathways, are monitored to understand the state of the underwater environment and the growth and health of underwater life through the intelligent analysis of real-time photographs taken underwater. However, the accuracy of the intelligent analysis results is greatly influenced by the underwater images’ quality; a complex imaging environment results in color casts and a loss of detail in the images obtained [1,2]. As a result, it is critical to achieve clarity and improve the details in underwater images.

Underwater image enhancement is developing rapidly via both traditional and deep learning. In traditional methods, Drews et al. [3], influenced by the dark channel prior (DCP [4]), proposed transmission estimation in underwater single images (UDCP), which does not take into account the effects of red channels but is prone to overexpression. Ma et al. [5] proposed the restoration of underwater images using a mix of improved dark channel prior and gray world methods; this new model improved the DCP and gray world theory to restore underwater images. Ancuti et al. [6] used multi-scale fusion to generate an image that was clear after white balance and gamma correction had been performed on the damaged image. To improve underwater image quality, Liang et al. [7] combined color correction based on attenuation maps with a detail retention and haze removal method based on multi-scale decomposition. Marques et al. [8] derived an effective atmospheric illumination model from local contrast information based on human observations and, from this model, they generated an enhanced image for highlighting details and an enhanced image for removing darkness. In turn, the underwater image is enhanced via multi-scale fusion. The interpretability of traditional methods is obvious, but the effect needs to be further improved.

In recent years, the application of deep learning methods in underwater image processing has become more and more prominent, especially deep learning methods based on genetic algorithms. For example, Li et al. [9] proposed Water-GAN to enhance underwater images. Synthetic underwater images are utilized as datasets for training the neural network to perform underwater image color correction. Fabbri et al. [10] suggested the enhancement of underwater imagery using generative adversarial networks. They first applied CycleGAN to paired images to create degraded underwater images. The underwater image pairs were then selected as datasets for further network training. Guo et al. [11] designed a multi-level intensive generative adversarial network, containing two multi-scale dense blocks that can correct color differences and enhance image details. Islam et al. [12] suggested fast underwater image enhancement to enhance the perception of images (FUnIE-GAN) based on U-Net, which improves image detail clarity by using residual connections in the generator. GAN-RS, a multi-branch discriminator proposed by Chen et al. [13], was developed to increase the quality of underwater images. However, numerous training parameters require careful tuning. If you train with incorrect parameters, the resulting images will produce artifacts. Huang et al. [14] proposed Semi-UIR, to enhance the model performance with a semi-supervised method and mean-teacher-based underwater image restoration model, by constructing a reliable bank and contrast learning. Compared with the traditional learning methods, a deep learning method can better solve the image color distortion problem and has a superior portability and learning ability in image processing.

**Figure 1:**
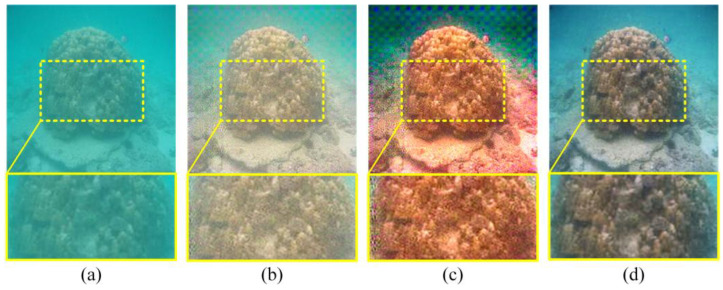
Image comparison: (**a**) degraded image, (**b**) FUnIE-GAN, (**c**) UDCP, and (**d**) ours; the image in the second row is the enlarged detail image of the yellow box in the first row.

The above methods focus on enhancing underwater images, as shown in Figure 1. However, their algorithm is not well-suited to intricate scenarios due to the lack of attention given to the color and data loss caused by the imaging environment. In addition, most methods that are available improve the network by increasing the network depth; however, this will result in problems such as gradients, training difficulties, and unstable parameters [15].

To solve the problems above, we propose the implementation of the Dense Residual Generative Adversarial Network (DRGAN). Here are the primary contributions:

(1) A multi-scale feature extraction module is proposed to extract image detail information and expand the receptive field.
(2) A dense residual block is proposed to fuse feature maps into clear images, not only fully utilizing all layers with local dense connections but also adding residual connections to reuse information.
(3) We combine multiple loss functions to facilitate the learning of the generator regarding the generation of clear images. The experimental results show that DRGAN outperforms the state-of-the-art methods in terms of qualitative and quantitative indicators.

The remainder of this work is structured as follows. Section 2 discusses related work, such as dense residual theory and GANs. Section 3 describes our proposed method in detail, and Section 4 presents and discusses the experimental results and analysis. Finally, Section 5 concludes the paper.

## 2. Related Work

### 2.1. Generative Adversarial Network

Generative adversarial networks are composed of two distinct neural networks: a generator and a discriminator [16]. In this paper, we employ a generator to produce a distinct image from the deteriorated image; the discriminator utilizes both the clear image and the generated image as the input, and it outputs the probability that the generated image is true. The generator and discriminator engage in an adversarial relationship during training to encourage the discriminator to accurately distinguish between genuine and counterfeit samples. In the end, we want the generator to produce images of high quality as a result of the network.

### 2.2. Residual Network

He et al. [17] suggested a residual network as a solution to the issue of numerous parameters decreasing due to the network’s excessive depth.

**Figure 2:**
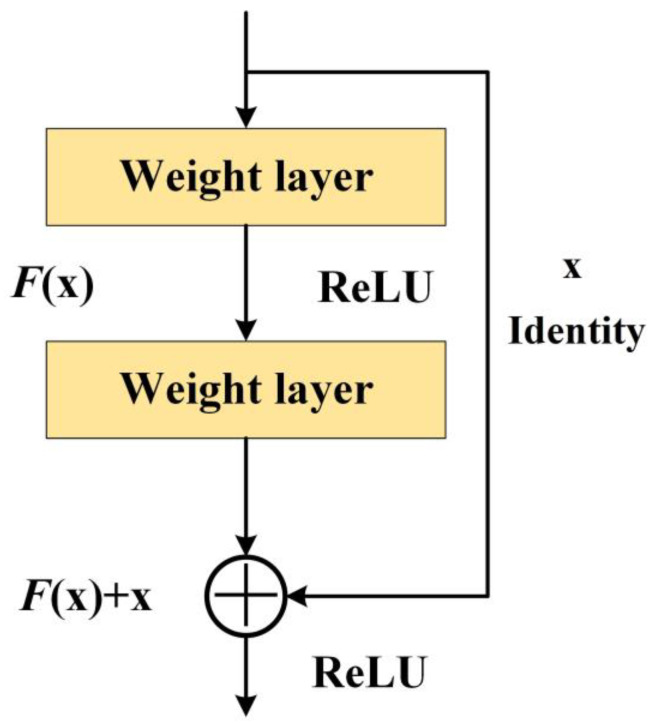
The residual network structure.

As shown in Figure 2, the addition of the details of the shallow layers to the subsequent deep layers allows the deep layers to focus on learning, avoids the loss of feature information, and prevents model degradation. Consequently, utilizing this technique on deep networks can address issues such as the escalation of gradients during training.

### 2.3. Densely Connected Convolutional Network

**Figure 3:**
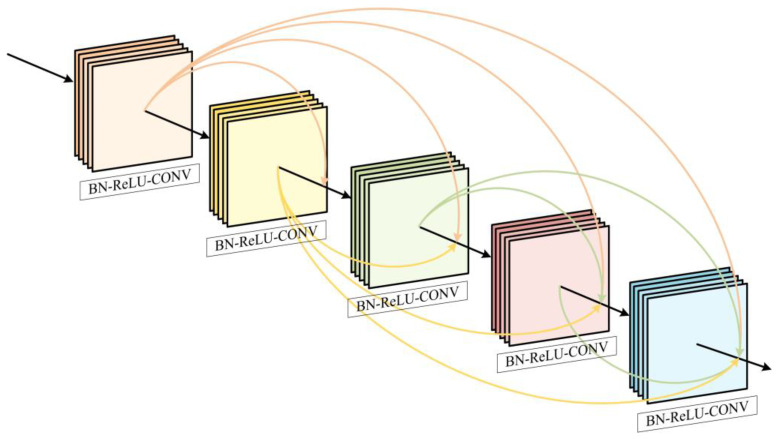
The diagram of our densely connected convolutional network structure. Different colors are used here to distinguish and emphasize the different nodes of the densely connected convolutional network structure.

The distinction between densely connected convolutional networks (DenseNets) [18] and residual networks lies in the fact that DenseNets facilitate the transmission of data between the various layers of the network and boost the number of links in each layer of the network, leading to improved feature reuse and a more powerful gradient propagation. As illustrated in Figure 3, the feature output of the preceding layer can be sent to all the following layers, and the transmission of feature information is improved via the linking of the layers in the network in pairs. Due to each layer being connected to all the previous layers when the gradient is back-propagated, the gradient is transferred to all the preceding layers in turn, a small number of convolutional kernels can still produce a substantial amount of feature information, and the preceding layers can fine-tune their parameters by taking advantage of the gradient data from the subsequent layers. Through this process, the issue of gradient disappearance is lessened, and the network’s training performance is enhanced.

The dense connections in Figure 3 can be represented as:

(1) $$X_{n}=H_{n}\left(\left[X_{1},X_{2},\ldots ,X_{n}\right]\right),$$

where $\left[X_{0},X_{1},\ldots ,X_{n-1}\right]$ is the output of the characteristic map.

## 3. Our Method

### 3.1. Generative Network

**Figure 4:**
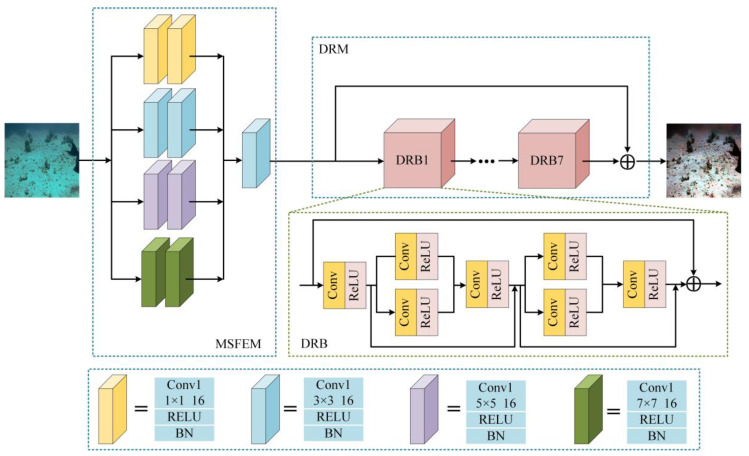
The diagram of our generative network structure, where different colors represent convolutional layers with different convolutional kernels.

Our generator includes a multi-scale feature extraction module (MSFEM) and dense residual block (DRB), facilitating the generation of crystal-clear underwater images. Using the yellow convolution unit in the MSFEM as a case in point, as demonstrated in Figure 4, Conv1 is a convolution with a stride of 1, the number of convolution kernels is 16, the ReLU is an activation function, and the BN is a representation of batch normalization.

**Figure 5:**
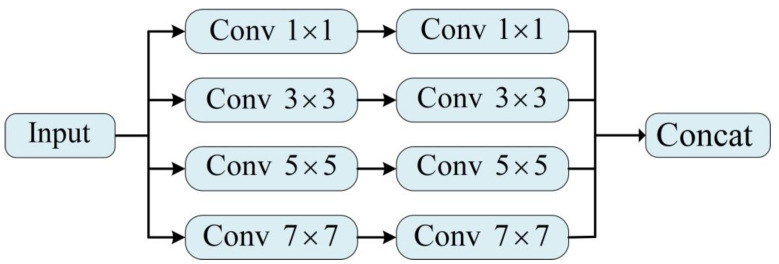
The diagram of our multi-scale feature extraction module.

As shown in Figure 5, 1×1 and 3×3 focus on extracting image detail information, the 5×5 and 7×7 convolution kernels can better extract the global features of the image, and each parallel convolution unit consists of two identical convolution kernels. By combining the above, MSFEM can extract different spatial features from the input image. We use concatenation to combine four feature maps to realize feature information fusion. To a certain extent, the loss of shallow details is avoided. The output result can be expressed as:

(2) $$F=\mathrm{Concat}(f_{1},f_{2},f_{3},f_{4}),$$

where F is the feature image processed by the module, and $f_{1}$, $f_{2}$, $f_{3}$, and $f_{4}$ are the feature images obtained by the four convolution units, respectively.

As illustrated in Figure 4, we designed a DRB composed of 3×3 convolution kernels. The goal of dense connections is to process as much information as possible from all layers, and the use of residual connections not only improves the utilization of information and ensures image integrity but also enables the next dense residual block to preprocess the information.

By combining residual and dense connections, the DRB ensures the correct transmission of feature information and reduces the computational complexity of the module. More importantly, compared with other networks, the network efficiency is improved without additional logistical costs, making feature information fusion more efficient. The result of the residual can be expressed as:

(3) $$Output=h(x_{1})+F(x_{1},W_{l}),$$

where Output is the output image of the module, $h(x_{1})$ is the direct mapping, $F(x_{1},W_{l})$ is the residual part, $x_{1}$ is the input, and $W_{l}$ is the convolution operation.

**Figure 6:**
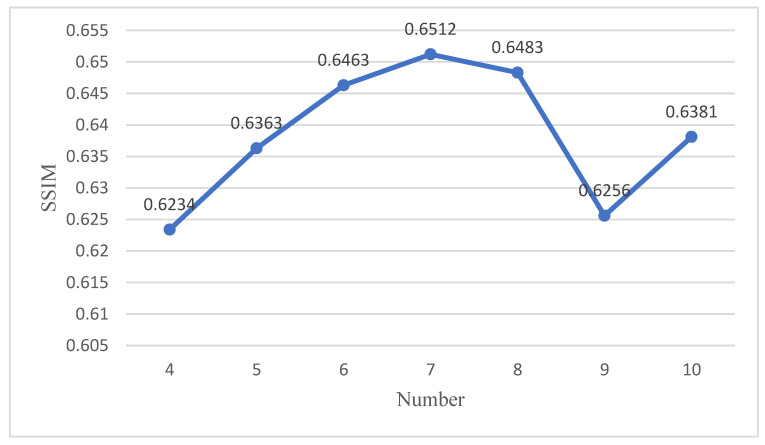
The number of dense residual block cycles.

Derived from the SSIM test results shown in Figure 6, the number of dense residual block cycles of the network is set to 7 to achieve the most desired outcome.

### 3.2. Discrimination Network

**Figure 7:**
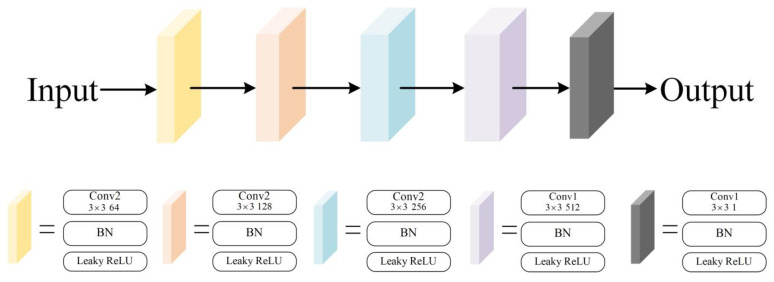
The diagram of our discriminator network structure.

The proposed discriminating network, as illustrated in Figure 7, is made up of five-layer convolution units, like the design of PatchGAN [19]. The convolution unit follows the Conv-BN-Leaky ReLU structure, and the step size of multiple units is set to 2 to increase the receptive field of the output characteristics. Due to the problem of neurons not being able to learn after the ReLU function enters the negative interval, we chose the leaky ReLU function in order to limit the appearance of silent neurons. The network uses the generated image and undistorted image as the inputs, and it outputs an image with a size of 30×30. The discrimination network operates on small-sized image blocks, which greatly reduces the number of parameters and amount of computation, while also alleviating the problem of slow convergence that is characteristic of the GAN. The leaky ReLU expression is shown in the formula:

(4) $$LeakyReLU=\left\{\begin{array}{l} x,x>0\\ \alpha x,x\leq 0 \end{array}\right.,$$

where α is a tiny constant used to maintain some negative axis values so that the information on the negative axis is not completely lost.

In the last layer, the sigmoid function is used to map the output pixel range to the undistorted image, which can clearly distinguish the authenticity of the created image and the undistorted image in a certain area. The function expression is as follows:

(5) $$f(x)=\frac{e^{-x}}{{\left(1+e^{-x}\right)}^{2}},$$

### 3.3. Loss Function

The adjustment of the network is facilitated by the linear combination of GAN loss and SSIM loss, as outlined below.

(1) GAN Loss

GAN loss functions are used to make the generated sample distribution as close to the true sample distribution as possible. The following is the definition of countermeasure loss:

(6) $$L_{\mathrm{GAN}}(G,D)=E_{X,Y}[\log D(Y)]+E_{X,Y}[\log(1-D(X,G(X,Z)))],$$

where X is the degraded image, Y is the undistorted image, E is the mathematical expectation, and Z denotes the random noise. To ensure that D recognizes the image produced by G as an undistorted image, G generates an image that conforms to the undistorted data distribution as much as possible.

(2) SSIM Loss

The structural similarity of the two images is measured using SSIM loss. SSIM loss functions similarly to the human visual system. It is sensitive to the perception of local structural changes and is conducive to enhancing the image’s texture details. SSIM loss is defined as:

(7) $$L_{\mathrm{SSIM}}(P)=1-\mathrm{SSIM}\left(\tilde{p}\right),$$

where P is the image block, and $\tilde{p}$ is the image block’s center pixel.

(3) Perceptual Loss

The parameters of the feature map in the trained convolutional neural network define the perceptual loss. The image details obtained after the function participates in the training are more realistic. The perceptual loss is defined as:

(8) $$L_{Z}=\frac{1}{W_{i,j}H_{i,j}}\sum_{x=1}^{W_{i,j}}\sum_{y=1}^{H_{i,j}}\left[\phi_{i,j}{\left(I_{Q}\right)}_{x,y}-\phi_{i,j}{\left(G\left(I_{S}\right)\right)}_{x,y}\right],$$

where $\phi_{i,j}$ is the characteristic diagram of the output of the j convolution layer before the i pooling layer in the pre-training VGG19 network, and $W_{i,j}$ and $H_{i,j}$ are the dimensions of the characteristic diagram. In this paper, i is taken as 4, and j is taken as 3; the ${\mathrm{VGG}}_{4,3}$ convolution characteristic diagram is selected to define the loss.

(4) Overall Loss

The function of the overall loss obtained via the linear combination of the three loss functions can effectively improve the robustness of the network and is defined as:

(9) $$L=\lambda_{1}*L_{\mathrm{GAN}}+\lambda_{2}*L_{\mathrm{SSIM}}+\lambda_{3}*L_{Z}.$$

After many experiments, $\lambda_{1}$ is taken as 1, $\lambda_{2}$ as 100, and $\lambda_{3}$ as 10.

## 4. Experiment

To verify the effectiveness of DRGAN, in this study, we firstly set the experimental details. Then, we compared DRGAN with different representative methods. These methods included Fusion [6], ICCB [20], L^2UWE [8], FUnIE-GAN [12] (replaced with FUnIE below), Semi-UIR [14], and UWCNN [21]. Finally, to validate the components of DRGAN, we performed ablation experiments. Furthermore, we conducted experiments such as feature point matching and edge detection to validate the usefulness of our approach in real-world applications.

### 4.1. Experimental Details

We conducted experiments on the Underwater ImageNet [10] dataset and RUIE [22] dataset, respectively. The details are as follows. (1) From the Underwater ImageNet dataset, we randomly selected 4000 pairs of images from underwater scenes for training and 2000 pairs for testing. (2) We exploited the trained model of the Underwater ImageNet dataset to test the RUIE dataset, which demonstrated the generalization ability of DRGAN. We trained DRGAN with Adam and set the training and test image size to 256 × 256 × 3, the batch size to 2, and the epoch to 50. TensorFlow was used as the deep learning framework on an Ubuntu 18.04 machine with 32 GB RAM and a GTX1070Ti (8 GB).

### 4.2. Subjective Evaluation

The color of the undistorted swatch image would be degraded because of the complex underwater imaging environment. Therefore, the color restoration impact of DRGAN could be efficiently tested through color recovery experiments on the color card [23].

**Figure 8:**
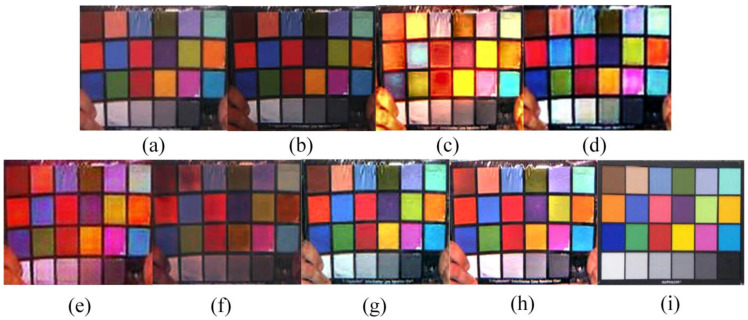
A qualitative comparison of the results of the color recovery experiment: (**a**) the degraded image, (**b**) Fusion, (**c**) ICCB, (**d**) L^2UWE, (**e**) FUnIE, (**f**) UWCNN, (**g**) Semi-UIR, (**h**) ours, and (**i**) the standard color card.

As can be seen in Figure 8, the Fusion method reduces the contrast between the yellow and pink color blocks, while deepening the overall hue of the color card picture, and the image processed via the ICCB algorithm suffers from a color distortion problem. Although the Semi-UIR algorithm can achieve color correction, the visual effect is negatively affected by the overall redness of the processed image. The problem of low discrimination is shown in the image that was processed via the L^2UWE algorithm, as evidenced by the dark purple and green color cards that are visually close to black. Overall, the color cards processed via the UWCNN algorithm suffer from poor color correction, as shown through the blueish hue. Overall, the FUnIE algorithm tends to make the color cards appear red during the experiment. On the contrary, our method achieves promising visual results with the color card images, especially when dealing with indistinguishable color patches (specifically black, purple, and dark green), validating the superiority of the color correction capability of our method.

**Figure 9:**
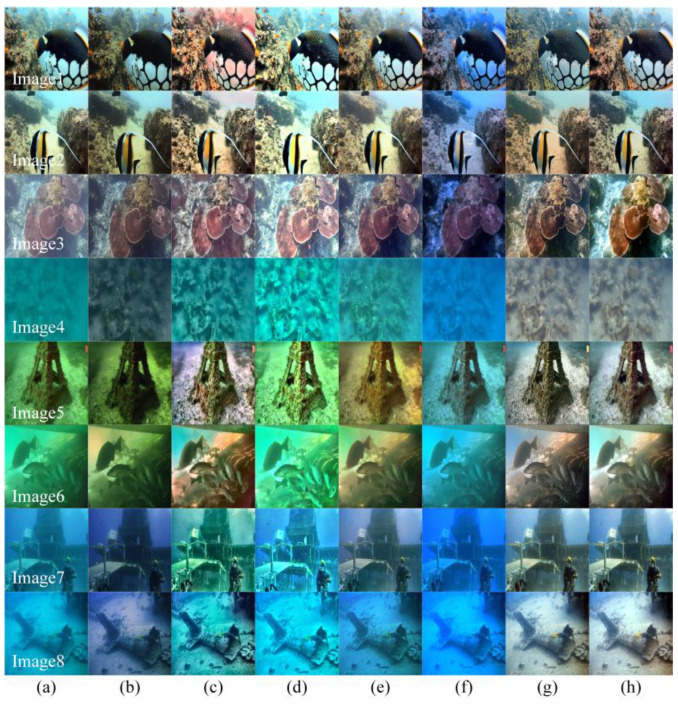
A qualitative comparison of the results of the complex underwater environment experiment, including our method and SOTA methods: (**a**) the degraded images, (**b**) Fusion, (**c**) ICCB, (**d**) L^2UWE, (**e**) FUnIE, (**f**) UWCNN, (**g**) Semi-UIR, and (**h**) ours.

Next, the method was applied to images from a complex underwater environment. The input image was affected by different degrees of color distortion, low brightness, and turbidity, resulting in various degradation phenomena. Figure 9 illustrates the processing results for each method. Images 1–2 are the normal degraded images, Images 3–4 are the atomized images, and Images 5–6 and Images 7–8 are green and blue partial images, respectively.

As can be seen in Figure 9, the Fusion algorithm fails to improve the sharpness and quality of low-brightness and color-distortion images. The ICCB algorithm has some success in improving the brightness and color correction, but the vividness of the image colors is greatly reduced. The L^2UWE algorithm fails to improve green, blue, and normal degraded images. Although the fogging problem can be mitigated, the generated image seems to have insufficient brightness. The FunIE algorithm can solve the problem of low brightness, but the problem of color distortion remains. The fogged image processed via FUnIE shows the problem of an obvious reddish tint, which is not consistent with the real image, and the image processed via the UWCNN algorithm cannot achieve a good visual effect due to the overall bluish color. Image processing using the Semi-UIR method achieves some success in defogging and color correction, but the overall brightness of the final image is low. In addition, as shown in Figure 5, Figure 6, Figure 7 and Figure 8, the ICCB method is unable to perform effective deblurring, as evidenced by the severe color distortion. On the contrary, the results of our method show brighter and clearer images compared to all the tested comparison algorithms. It was found that the algorithm can address degradation in complex underwater environments (off-color, low brightness, high turbidity, etc.) and that it exhibits a strong robustness. It was determined through subjective evaluation that our method produces better-clarity results for images with different degrees of degradation compared to other, similar new methods.

### 4.3. Objective Evaluation

The image quality when applying our method was further evaluated through five objective evaluation indexes: UCIQE, UIQM, SSIM, PSNR, and CIEDE2000.

(1) The underwater color image quality evaluation index [24] (UCIQE) is proportional to the underwater picture quality, and the formula for calculating the index is as follows:
  (10) $$UCIQE=c_{1}\times \sigma_{c}+c_{2}\times con_{l}+c_{3}\times \mu_{s},$$
  where $\sigma_{c}$ is the chromaticity standard deviation, $con_{l}$ represents the contrast in brightness, $\mu_{s}$ represents the average value of saturation, and $c_{1}$, $c_{2}$, and $c_{3}$ are weighting coefficients.
(2) The underwater image quality measurement [25] (UIQM) is a quality-evaluated indicator of non-reference underwater images based on human visual system excitation. The calculation formula is as follows:
  (11) $$UIQM=c_{1}\times UICM+c_{2}\times UISM+c_{3}\times UIConM,$$
  where $c_{1}$ is set to 0.0282, $c_{2}$ is set to 0.2953, and $c_{3}$ is set to 3.5735. The underwater image quality measurement is a linear combination of the underwater image colorfulness measure (UICM), underwater image sharpness measure (UISM), and underwater image contrast measure (UICONM). The higher the UIQM, the better the image’s color balance, sharpness, and contrast.
(3) The structural similarity index measurement [26] (SSIM) is an index for determining how similar the two images are. When two images, x and y, are given, the calculation formula is:
  (12) $$SSIM(x,y)=\frac{\left(2\mu_{x}\mu_{y}+c_{1})(2\sigma_{xy}+c_{2}\right)}{\left(\mu_{x}^{2}+\mu_{y}^{2}+c_{1})(\sigma_{x}^{2}+\sigma_{y}^{2}+c_{2}\right)},$$
  where $\mu_{x}$ and $\mu_{y}$ are the average of x and y, respectively; $\sigma_{x}^{2},\sigma_{y}^{2}$ are the variance of x and y; and $c_{i}=k_{i}L,(i=1,2)$ is a constant to maintain stability. $\sigma_{xy}$ is the covariance of x and y; $k_{1}=0.01,k_{2}=0.03$.
(4) The peak signal-to-noise ratio (PSNR) is an index to measure image quality. The calculation formula for the mean square error (MSE) is:
  (13) $$MSE=\frac{1}{mn}\sum_{i=0}^{m-1}\sum_{j=0}^{n-1}{\left[I_{o}(i,j)-I_{p}\right]}^{2},$$
  where two images, $I_{o},I_{p}$, are compared. The PSNR is obtained through the MSE, and the calculation formula is:
  (14) $$PSNR=10\log_{10}(\frac{{\left(2^{n}-1\right)}^{2}}{MSE})$$
(5) The CIEDE2000 evaluation index [27], which has a range of [0, 100], measures the color changes between the standard color card and each processed color block. The color differences are reduced when the index decreases. For the evaluation in Figure 8, we used the CIEDE2000 evaluation index. Table 1 displays the results.

Comparing the data in Table 1, we can see that, like DRGAN, FUnIE, and Semi-UIR both achieve good results, and FUnIE adds residual connections to the generator to enhance the network performance. DRGAN’s CIEDE2000 average result is the lowest, showing that our technique performs better in terms of color recovery.

We used UCIQE to evaluate the images in Figure 9, and the results are shown in Table 2. The results show that the average value of the DRGAN algorithm is higher than that of other algorithms. For Images 1 and Image 2, the DRGAN UCIQE was lower than that of L^2UWE because the original image was less degradable, and Semi-UIR recovery was better than DRGAN enhancement. However, ICCB, with a higher UCIQE, showed significant color aberration in Image 6 and unnatural color restoration in Image 8.

The UIQM results from Figure 9 are shown in Table 3; our average UIQM for DRGAN is higher than for the other algorithms. The light degradation of Image 1 and Image 3 leads to a higher UIQM in the ICCB restoration algorithm than the enhancement effect of DRGAN, and when processed via FUnIE, Image 2 has a yellow color cast. Although Semi-UIR achieves good enhancement results, it is not thorough enough in detail processing, as shown in Image 8.

As the RUIE dataset has no ground truth, we chose the UIQE and UIQM metrics when comparing with other algorithms, and we used the UCIQE, UIQM, SSIM, and PSNR metrics on the Underwater ImageNet dataset. The test results using the Underwater ImageNet dataset and RUIE dataset are shown in Table 4 and Table 5.

We verified the effectiveness of DRGAN on the Underwater ImageNet dataset and applied the model trained on the Underwater ImageNet dataset to the RUIE dataset. In the Underwater ImageNet dataset, DRGAN’s PSRN, UIQM, and UCIQE outperformed the other algorithms, indicating that the DRGAN enhancement results are closer to the real images. On the RUIE dataset, on average, DRGAN also achieved better results. While FUnIE also adds residual connections, it is only in the Green and Atomization environments in the RUIE dataset that the SSIM indicators for the Underwater ImageNet dataset are better than those of DRGAN. From the above, it can be concluded that the addition of dense and residual connections in DRGAN has a better performance-enhancing effect and leads to a better generalization ability.

### 4.4. Ablation Study

We conducted module ablation experiments using the Underwater ImageNet dataset. Firstly, we evaluated the images processed via different modules using PSNR, SSIM, UCIQE, and UIQM. Table 6 shows the objective metric scores for the ablation experiments, where w/o MSFEM denotes the removal of the multi-scale feature extraction module, w/o DRB denotes the removal of the dense residual block, w/o RES denotes the removal of residual connectivity in the DRB, and w/o DEN denotes the removal of dense connectivity in the DRB.

Table 6 shows the performance w/o MSFEM and w/o DRB on UCIQ, where it can be seen that the performance w/o MSFEM on UIQM is higher than that w/o DRB. Removing both the dense connections and the residual connections influences the model performance. This result fully demonstrates the importance of the two modules we adopted, MSFEM and DRB, for the overall performance of the network.

**Figure 10:**
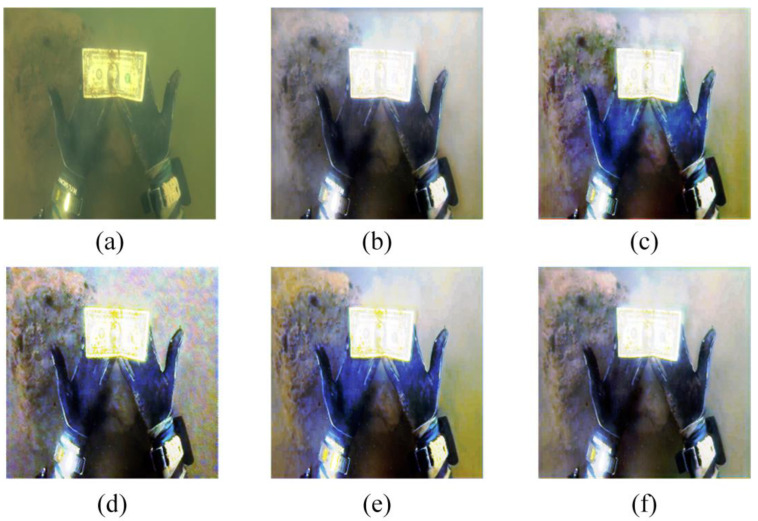
Image showing the ablation experiment results: (**a**) the degraded image, (**b**) w/o MSFEM, (**c**) w/o RES, (**d**) w/o DEN, (**e**) w/o DRB, and (**f**) DRGAN.

Then, we randomly selected an image for subjective comparison. Figure 10 shows that the image processed w/o DRB has artifacts and is accompanied by a yellow color cast, while the image processed w/o MSFEM is subjectively better than that w/o DRB, but still has a small amount of color cast. The image enhanced via the full processing model is the best and the most visually natural. Figure 10 also demonstrates that the image color recovery is poor, and there are artifacts, after the removal of the residual connections in the dense residual block. On the contrary, after the removal of the dense connections in the dense residual block, the image is over-enhanced.

### 4.5. Additional Experiments

**Figure 11:**
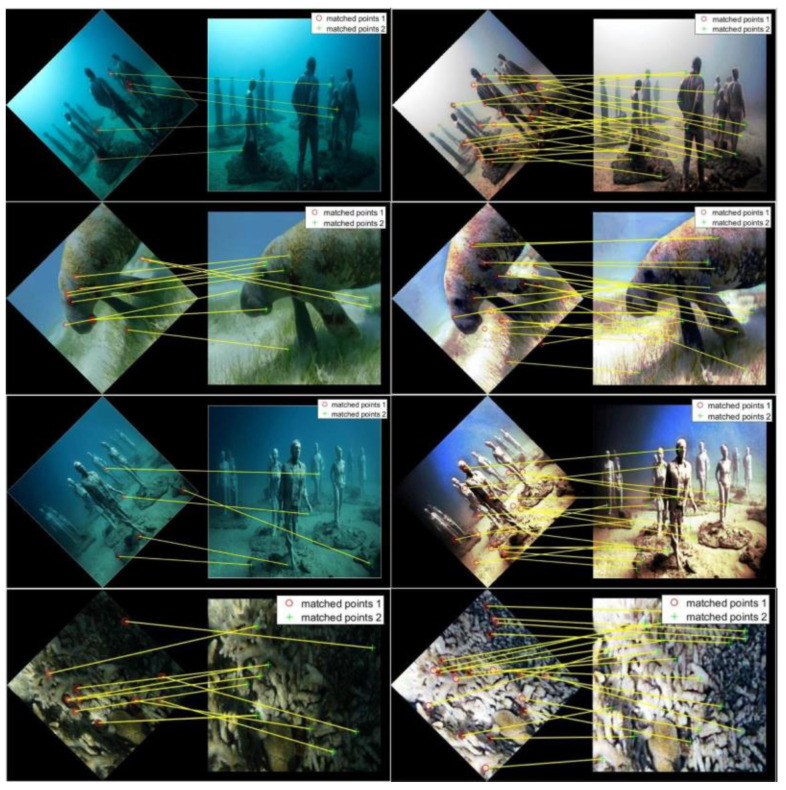
Feature point matching: (**left**) the degraded image, (**right**) ours.

**Figure 12:**
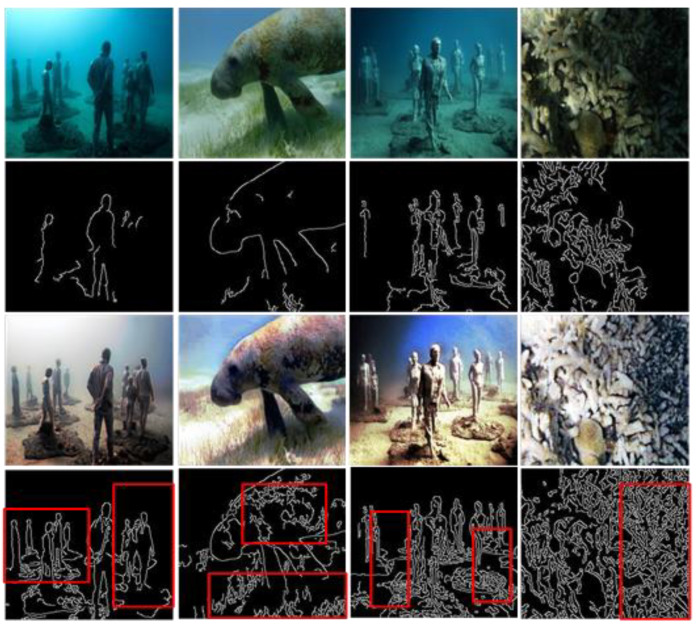
The Canny operator results. The images in the first and third rows represent degraded and enhanced images following our algorithm, while the images in the second and fourth rows represent the outcomes of the Canny operator detection output. Additionally, the red box line in the fourth row illustrates the more comprehensive image information that can be acquired through the processing of our algorithm.

Less image feature information makes underwater image detection more challenging. As shown in Figure 11 and Figure 12, several images were selected for surf feature point matching and Canny operator experiments, which verified that our method can enhance edges and other feature information in underwater images.

Figure 11 shows the results from the surf feature point matching; it can be seen that the processed image has significantly more feature points than the original underwater image. These experiments show that the proposed algorithm successfully enriches the characteristics of underwater images, making the subsequent information processing much easier.

Figure 12 shows the results of the Canny operator; after the processing in this method, more details of the image can be added (such as coral patterns, etc.). Compared with the degraded images, DRGAN can clearly show the contour information of the picture. This makes the detection and tracking of features of interest via underwater robots a much less taxing endeavor.

## 5. Conclusions

In this paper, we propose DRGAN as a means of enhancing underwater images, drawing inspiration from ResNet and DenseNet. Through the utilization of a multi-scale feature extraction module and a dense residual block in the generator, multi-scale feature information is integrated. The incorporation of these multi-stage features broadens the receptive field and safeguards against any decline in network performance due to gradient disappearance. Additionally, DRGAN optimizes network utilization by leveraging the benefits of both residual and dense connections. It is worth mentioning that the generator’s computational efficiency has been enhanced in comparison to networks that solely rely on dense blocks. We employ a discriminator akin to PatchGAN for adversarial training, and this augments the generator’s ability to sharpen images. The findings from the experiments conducted on intricate underwater scenes indicate that DRGAN greatly enhances the quality of images in comparison to various renowned techniques. In the future, we plan to use the proposed method in other areas of marine engineering, such as object recognition and detection within wider underwater scenes.

## Figures and Tables

**Table 1:** The evaluation results for CIEDE2000; the black bold font represents the best data.

Method	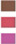	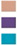	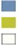	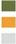	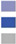	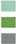	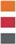	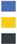	Avg
Fusion	26.34	33.74	33.02	32.27	35.44	22.70	29.93	19.05	27.23
	29.39	19.34	38.37	39.61	13.52	39.20	19.63	40.03	
	25.31	28.82	21.08	22.20	26.48	30.73	19.30	8.02	
ICCB	26.18	29.71	40.49	35.84	27.65	32.60	**11.15**	48.19	24.04
	21.58	40.94	14.31	20.06	28.25	29.16	8.77	**9.24**	
	16.10	31.84	11.43	17.52	27.40	21.04	**14.57**	12.85	
L^2UWE	18.20	17.17	16.06	22.58	13.12	11.54	20.94	15.96	16.35
	17.20	**7.34**	25.42	27.60	10.04	27.67	7.86	25.79	
	8.20	11.54	15.30	14.80	14.22	18.52	14.89	10.67	
FUnIE	**17.91**	18.17	15.06	23.58	12.52	10.64	21.28	16.23	16.82
	17.80	8.36	27.45	28.20	9.07	29.65	**6.82**	27.79	
	8.30	11.94	15.15	15.50	15.19	20.47	15.88	10.63	
UWCNN	23.11	40.23	31.11	32.63	37.51	38.10	33.11	17.54	28.40
	24.50	16.21	46.90	54.85	18.02	38.71	15.21	16.21	
	23.61	26.54	41.91	18.11	37.60	31.74	15.14	**3.80**	
Semi-UIR	21.80	10.90	13.13	15.81	12.24	**10.51**	24.82	13.91	14.14
	**17.21**	14.92	**10.62**	17.94	7.94	**2.72**	15.20	17.50	
	12.91	9.41	15.60	9.22	**7.3**	26.21	21.91	10.51	
Ours	19.11	**9.27**	**8.34**	**13.90**	**9.85**	13.41	22.80	**11.40**	**13.79**
	39.34	12.91	11.2	**16.81**	**3.90**	15.61	7.2	13.51	
	**2.71**	**7.10**	**5.30**	**8.10**	37.63	**13.71**	15.10	13.81	

**Table 2:** The quantitative comparison using the UCIQE dataset; the black bold font represents the best data.

No.	Fusion	ICCB	L^2UWE	FUnIE	UWCNN	Semi-UIR	Ours
1	0.5970	0.5579	**0.6057**	0.5791	0.5272	0.5891	0.5982
2	0.5024	0.5646	**0.6063**	0.6012	0.5258	0.5746	0.6012
3	0.5184	0.5101	0.5331	0.5331	0.5118	0.6316	**0.6470**
4	0.4361	0.3777	0.4971	0.4971	0.5189	0.6198	**0.6200**
5	0.5504	**0.6930**	0.6863	0.5359	0.5006	0.6066	0.6012
6	0.5403	0.5572	0.6826	0.6741	0.5920	0.5950	**0.6976**
7	0.4914	0.5312	0.5404	0.5404	0.4605	0.5736	**0.5821**
8	0.4770	0.5548	0.5694	0.5694	0.4563	**0.7085**	0.6072
Average	0.5117	0.5433	0.5901	0.5674	0.5083	0.6124	**0.6218**

**Table 3:** The quantitative comparison using the UIQM dataset; the black bold font represents the best data.

No.	Fusion	ICCB	L^2UWE	FUnIE	UWCNN	Semi-UIR	Ours
1	4.8645	**5.0569**	4.5500	4.2465	4.4736	4.8277	4.8126
2	4.7372	5.0987	5.1155	**5.2598**	4.4404	4.7455	4.8373
3	5.4121	**5.8937**	4.9376	3.7809	4.2900	4.7741	4.3680
4	4.0254	4.0917	3.3799	4.0478	4.1496	4.6246	**4.6264**
5	4.3309	4.7002	3.5894	4.0904	4.4431	4.6995	**4.8586**
6	3.8358	3.9490	3.0448	4.6219	4.2285	4.3388	**4.9539**
7	3.5990	1.6884	3.2024	3.8366	2.4250	3.6705	**4.4406**
8	3.5839	2.8450	2.3827	2.2734	1.8241	3.8842	**4.5264**
Average	4.2986	4.1654	3.7752	4.0196	3.7842	4.4706	**4.8029**

**Table 4:** The quantitative comparison using the Underwater ImageNet dataset; the black bold font represents the best data.

Method	SSIM	PSNR	UIQM	UCIQE
Fusion	0.636	25.794	4.266	0.541
ICCB	0.748	31.341	4.376	0.608
L^2UWE	0.746	26.387	4.338	0.601
FUnIE	0.774	34.257	4.995	0.588
UWCNN	0.648	28.868	4.463	0.507
Semi-UIR	**0.827**	31.033	5.037	0.592
Ours	0.661	**37.592**	**5.087**	**0.630**

**Table 5:** The quantitative comparison using RUIE. (M) and (E) represent UIQM and UCIQE, respectively. The black bold font represents the best data.

Method	Blue		Green		Atomization		Normal		Average	
	(M)	(E)	(M)	(E)	(M)	(E)	(M)	(E)	(M)	(E)
Fusion	3.770	0.580	4.400	0.533	4.516	0.495	4.701	0.587	4.350	0.545
ICCB	5.422	0.588	4.272	0.568	4.742	0.582	4.450	0.610	4.698	0.585
L^2UWE	4.394	0.575	4.606	0.578	**5.336**	0.519	5.273	0.583	5.003	0.545
FUnIE	3.836	0.628	4.165	0.602	4.286	0.599	4.395	0.617	4.170	0.611
UWCNN	4.189	0.564	4.147	0.524	4.096	0.521	5.058	0.590	4.339	0.547
Semi-UIR	5.059	0.576	**4.973**	0.586	5.069	0.576	5.47	0.629	5.101	0.597
Ours	**5.680**	**0.653**	4.830	**0.634**	4.767	**0.640**	**5.870**	**0.640**	**5.360**	**0.641**

**Table 6:** The ablation experiment results for different variants of DRGAN; w/o refers to without. The black bold font represents the best data.

Modules	Baselines	PSNR	SSIM	UCIQE	UIQM
MSFEM	w/o MSFEM	34.372	0.653	0.615	4.734
DRB	w/o RES	35.132	0.651	0.619	4.833
	w/o DEN	34.876	0.651	0.614	4.791
	w/o DRB	32.195	0.649	0.609	4.592
DRGAN	**full model**	**37.592**	**0.661**	**0.630**	**5.087**

## Data Availability

The underwater image data that support the findings of this study are openly available at https://irvlab.cs.umn.edu/resources/euvp-dataset (accessed on 30 May 2022) and https://github.com/dlut-dimt/Realworld-Underwater-Image-Enhancement-RUIE-Benchmark (accessed on 19 January 2022).
